# Feeding Strategies for Adapting Lake Sturgeon (*Acipenser fulvescens*) Larvae to Formulated Diets at Early Life Stages

**DOI:** 10.3390/ani12223128

**Published:** 2022-11-13

**Authors:** Seunghyung Lee, Shaowei Zhai, Dong-Fang Deng, Yuquan Li, Patrick Christopher Blaufuss, Bradley T. Eggold, Fred Binkowski

**Affiliations:** 1 School of Freshwater Sciences, University of Wisconsin-Milwaukee, Milwaukee, WI 53204, USA; 2 Major in Aquaculture and Applied Life Sciences, Division of Fisheries Life Sciences, Pukyong National University, 45 Yongso-ro, Nam-gu, Busan 48513, Korea; 3 Great Lakes Research Facility, Department of Natural Resources, 600 E. Greenfield Avenue, Milwaukee, WI 53204, USA

**Keywords:** *Artemia*, combined feeding, growth, larval fish culture, larval feed, survival, stock enhancement

## Abstract

**Simple Summary:**

Failure of larvae and juveniles to transition from live feed to prepared diets is a common cause of significant mortality in many fish species, including lake sturgeon (*Acipenser fulvescens*). Our study investigated feeding strategies that adapt lake sturgeon transition to formulated diets. The results showed that co-feeding formulated diets with live feed for periods of 3 or 4 weeks can improve growth and survival during this transition. Our finding also suggested that introducing formulated diets early possesses a potential to improve tolerance to environmental hypoxia, which may be due to balanced nutrient profiles.

**Abstract:**

Cost-effective feeding management is required to support conservation hatcheries for lake sturgeon (*Acipenser fulvescens*), an ecologically important species in the Great Lakes region. This study investigated an approach to transition lake sturgeon larvae from live feed (*Artemia*) to formulated feed and its effect on growth performance, survival, and response to acute hypoxia stress. The first experiment showed that sturgeon had similar (*p* > 0.05) growth and survival when fed *Artemia* or the combined feeding of *Artemia* with the commercial diet (crude protein, 551 g/kg diet). Feeding solely on the commercial or lab-made (crude protein, 491 g/kg diet) diet significantly reduced growth and survival (*p* < 0.05). In the second experiment, the growth performance of sturgeon (14 days post-hatch, DPH) fed with either *Artemia* only or combined feeding different feeding durations of two, three, and four weeks followed by a complete transition to the commercial diet. At the end of six weeks, the 3- and 4-week combined feeding periods resulted in significantly higher body weight and survival compared to the 2-week combined and the *Artemia* only feeding treatments. In the last experiment, sturgeons (27 DPH) were fed only with *Artemia* or combined feeding of *Artemia* with the commercial diet for four weeks followed by the complete transition to the commercial diet for two weeks. Eighteen fish from each treatment were investigated the response to acute hypoxic conditions (gradual decrease in dissolved oxygen level from 8 to 2.3 mg/L at the rate of 1 mg/L per hour). When the dissolved oxygen was between 3 and 4 mg/L, the mortality rate of the combination-fed sturgeon (11.7%) was significantly lower than those fed only *Artemia* (83.3%). These results clearly demonstrate that a commercial diet can partially replace *Artemia* at early life stages to improve growth, survival, and hypoxia tolerance and thus its co-feeding with *Artemia* is recommended.

## 1. Introduction

Lake sturgeon (*Acipenser fulvescens*) are ecologically important species of fish in the Laurentian Great Lakes and Mississippi River basins and are currently listed as a either species of special concern or threatened in different states of the Great Lake Region [1,2,3]. The total population of lake sturgeon was estimated to be more than fifteen million at its peak, but it is now reduced to less than 1% of the historic population. Different stressors including overfishing, habitat deterioration, reduced water quality, climate change, and invasive species have contributed to lake sturgeon extirpation in different regions [4,5]. Maturing at a late age (12–15 years for males and 18–27 years for females), it has a prolonged reproductive cycle [6]. With this special life history, a long-term management plan is needed to establish sufficient juvenile and adult fish to reach an adequate population restoration [7]. The stocking hatchery-reared juvenile sturgeon is one strategy that can enhance the recovery of lake sturgeon populations [8].

Many have explored feeding strategies to improve culture success in lake sturgeon hatcheries [9,10,11,12,13]. Overall, live *Artemia* remains an essential live feed for maintaining good growth and survival during the early life stages. Bauman et al. [10] reported that partial replacement of *Artemia* by formulated feed could maintain high survival, but reduced growth appeared within two weeks of feeding. In contrast to larval fish, juvenile lake sturgeon (56 days post-hatch: DPH) that had been weaned to formulated diet obtain better growth and have higher nutrient retention than those fed with bloodworm [12]. For the grow-out lake sturgeon, the formulated feed could also support good growth and survival observed under lab conditions [13]. This suggests that lake sturgeon can be raised on formulated feed if a proper weaning process is implemented during the larval stage. Thus, the objective of this study is to investigate feeding strategies to manage lake sturgeon weaning to formulated feed. The ultimate goal is to reduce labor and feed costs associated with juvenile production and produce healthy fish to support its population restoration.

## 2. Materials and Methods

Three feeding experiments were conducted using the same culture system at the School of Freshwater Sciences, University of Wisconsin-Milwaukee. In the first experiment (Feeding trial I), a 4-week feeding trial was conducted to compare the effect of feeding *Artemia*, a commercial diet, a lab-made diet, or a combination of *Artemia* with either formulated diet on the growth performance of lake sturgeon larvae. In the second experiment (Feeding trial II), a 6-week feeding trial was conducted to investigate the impact of *Artemia* or combined feeding (*Artemia* and a commercial diet) on the growth and survival of lake sturgeon weaned to the commercial feed only at different points (2, 3, and 4 weeks). In the third experiment (Feeding trial III), a 6-week feeding trial was conducted to compare the tolerance to hypoxia of lake sturgeon fed with *Artemia* or combined feed (*Artemia* and a commercial feed) for four weeks before being weaned to the commercial diet only. The experiments followed an animal care protocol approved by the Institute Animal Care and Use Committee (15-16 #60) at the University of Wisconsin-Milwaukee.

Seven-DPH larvae were stocked in circular fiberglass tanks (86 cm diameter, 61 cm height, 260 L water volume) supplied with flow-through degassed water at a rate of 5 L/min. Water temperature was gradually increased from 18 °C at stocking to 22 °C (0.5 °C increment per day). Dissolved oxygen was measured daily (YSI Pro1020, YSI Life Sciences, Yellow Springs, OH, USA) and was maintained at >8 mg/L throughout the rearing period. Once excretion of the melanin plugs from spiral valves was observed (11-12 DPH), a commercial feed (Otohime B1, Marubeni Nisshin Feed Co., LTD, Tokyo, Japan) and newly hatched *Artemia* nauplii (Great Salt Lake strain, Artemia International LLC, Fairview, TX, USA) were provided daily for acclimating the larvae to exogenous feeding. The *Artemia* nauplii were prepared by incubating decapsulated *Artemia* cysts (Great Salt Lake, Artemia International LLC, Fairview, Texas, USA). Decapsulation was performed according to the methodology described in Sorgellos et al. [14]. Cysts were hatched in 25 g/L salinity water at 28 °C. *Artemia* nauplii (incubated for 24–32) were collected to feed the larvae 6 times daily at 8:30, 10:00, 11:30, 13:00, 14:30, and 16:00 h.

### 2.1. Feeding Trial I

Two thousand and one hundred 14-DPH larvae (33 ± 4 mg; mean ± SD) were distributed into 21 polypropylene circular tanks (45 cm diameter, 50 cm height, 60 L water volume), with 100 larvae per tank, supplied with flow-through degassed water (flow rate: ca. 0.9 L/min) for acclimation of the larvae to the experimental set-up. During the one-week acclimation period, commercial feed (Otohime B1, Marubeni Nisshin Feed Co., LTD, Tokyo, Japan) was provided at a feeding rate of 13% body weight per day (BW/d) using 24 h automatic feeders (Lifegard Automatic Fish Feeder, Lifegard Aquatics, Cerritos, California, USA). *Artemia* nauplii were also fed to the larvae at the same rate (based on the estimation of the dry weight of the nauplii) six times per day (8:30, 10:00, 11:30, 13:00, 14:30, 16:00 h). The overall feeding rate, 26% BW/d, was adopted from a previous study conducted on white sturgeon during a similar life stage [15].

At the end of the acclimation period, all larvae were pooled into a large tank, and larvae (21-DPH, 62 ± 4 mg) were re-distributed into 15 experimental tanks at 75 larvae per tank. The tanks were randomly assigned to 5 diets with 3 replicated tanks per treatment (Table 1): A, *Artemia* nauplii; B, commercial larval feed (Otohime B1); L, a lab-made diet; A + B; and A + L. The lab-made diet was formulated to contain (g/1000 g): casein (280), gelatin (70), squid meal (60), wheat gluten (10), egg white (80), dextrin (113.3), wheat starch (60), a 1:1:1 mixture of soy oil: fish oil: lard (80), lecithin (60), cholesterol (1.2), sodium alginate (20), carboxyl methylcellulose (10), canthaxanthin (1), ascorbyl palmitate (0.5), choline chloride (0.5), glucosamine (1.5), potassium phosphate (10), sodium phosphate (5), Haematococcus (algae) powder (20), vitamin premix (15), mineral premix (1.5), and betaine hydrochloride (1.5). All ingredients were obtained from Sigma-Aldrich, Inc., St. Louis, MO, USA) except vitamin and mineral premixes, which were provided by the Bozeman Fish Technology Center (Bozeman, MT, USA) and shared the same formulation as that in Sealey et al. [16]. The algae powder was from Cyanotech Corporation, Kona Hawaii, USA. The lab-made diet was processed following a cold extruding method described by Jiang et al. [17]. Briefly, all dry ingredients were fully mixed to obtain a homogenous mixture before the oil and lecithin were added for further mixing. Then, the mixture was mixed with warm deionized water (80 °C) before extruding into 2 mm strands, which were post-cooked at 80 °C for 15 min to improve binding. The feed was air dried overnight at room temperature and then ground and sieved to a size suitable for the larvae depending on the fish sizes. The nutritional composition of test diets is presented in Table 2.

The feeding for treatment B and L only last for one week and the rest treatments lasted for 4 weeks. During the first week of the trial, high mortality (18–20%) was observed in the larvae fed the formulated feed only (B and L diets). In addition, the remaining larvae showed a very poor response to feeding. Thus, the two treatments were terminated at the end of the first week. The remaining treatments were continued until the end of the 4-week feeding trial. The newly hatched *Artemia* nauplii were provided four times daily at 8:30, 11:00, 13:30, and 16:00 h. The prepared diets were loaded into the same 24 h automatic feeder described previously and dispensed feed 8 times daily. The central standpipe was wrapped with a 1 mm^2^-opening nylon mesh screen and was cleaned daily. Tanks were siphoned out daily before the first feeding of the day. During the 4-week feeding trial, all larvae in each tank were batch-weighed weekly, and then the feeding rate was adjusted accordingly. The larvae were not fed for 20 h prior to weighing to reduce any stress caused by handling. The feeding rate was 20%, 7.5%, 6.5%, and 4.8% BW/d for the first, second, third, and last weeks of the feeding trial, respectively. The feeding rates were adopted from a previous study conducted on white sturgeon larvae [15] because relevant information on an optimum feeding rate for lake sturgeon at this life stage was not available.

Water temperature was measured twice daily in the morning and afternoon and was maintained at 21.8–23.5 °C during the trial. Dissolved oxygen was measured daily using a meter (YSI Pro1020, YSI Life Sciences, Yellow Springs) and was maintained at >8 mg/L throughout the trial. Total ammonia nitrogen and pH were measured weekly, using a test kit (Ammonia Test Strips, Hach, Loveland, CO, USA) and a pH meter (YSI Pro1020, YSI Life Sciences, Yellow Springs), respectively, and their levels were maintained at <0.08 mg/L and 7.5–7.6, respectively. The photoperiod was maintained at 12L:12D (12 h light and 12 h dark) throughout the trial.

At the end of the 4-week feeding trial, all fish in each tank were batch-weighed to obtain growth performance indices: weight gain, feed conversion ratio, condition factor, and protein efficiency ratio. These measurements were calculated using the following equations:

Weight gain (WG, %) = 100 × (final body weight (mg) − initial body weight (mg))/initial body weight (mg)

Feed conversion ratio (FCR) = dry feed weight provided (mg)/wet weight gain (mg)

Condition factor (CF) = 100 × Body weight (mg)/total body length (mm)^3^

Protein efficiency ratio (PER) = Fish weight gain (mg)/protein provided (mg)

Prior to weighing and sampling, the feed was withheld from fish for 20 h. After the weighing, all fish from each tank were euthanized with an overdose (500 mg/L) of MS-222 (Western Chemical, Ferndale, WA, USA) and rinsed with deionized water for sample collection. Ten fish per tank were collected for the assays of RNA/DNA ratio in the whole fish, and 19–25 fish per tank were used for measuring individual body weight and total length. The latter group of fish was also used for the analysis of whole-body proximate composition. All the above samples were snap-frozen in liquid nitrogen and stored at −80 °C until analysis.

RNA/DNA ratio is widely used as an indicator of the growth and nutritional status of fishes [18,19]. RNA/DNA ratio in the whole larva was measured following the protocol established for larval fish, using a fluorometric method [20]. Briefly, 10 pooled larvae from each tank were pulverized into powder using a liquid nitrogen-cooled mortar and pestle. Approximately 20 mg of each of the ground samples was mixed with 150 µL of 1% sarcosil Tris-EDTA buffer solution (STEB: 1% N-lauroylsarcosine (*w*/*v*), 5 mM Tris-HCl, 0.5 mM EDTA, pH 7.5) for 1 h at room temperature at 1200 rpm using a mixer (Thermomixer R, Eppendorf, Hamburg, Germany). Then, 1.35 mL of Tris-EDTA buffer solution was added to the above mixture, which was mixed 40 times by manual inversion. The samples were then centrifuged at 14,000 rpm for 15 min at room temperature, and the supernatant collected. DNA (originated from calf thymus; Product Code: D4764, Sigma-Aldrich, St. Louis, MO, USA) and RNA (16S- and 23S-ribosomal originated from *E. coli* MRE 600; Product Code: 10206938001, Roche Diagnostics, Indianapolis, IN, USA) standards were used to develop a calibration standard curve for estimation of DNA and RNA concentrations. The first reading (total concentration of RNA and DNA) was recorded at 525 nm excitation wavelength and 600 nm emission wavelength using a microplate fluorescence reader (Synergy H4 Hybrid Reader, BioTek, Winooski, VT, USA). Then, RNase (from bovine pancreas; Product Code: R6513, Sigma-Aldrich) solution (20 U/mL) was added to degrade the RNA to obtain a second reading of DNA concentration. Concentrations (µg) were calculated based on the fluorescent readings and the standard calibration curves, with the concentration of RNA calculated by subtracting the concentration of DNA from the total concentration.

The proximate composition of feed and whole larvae was determined following the methods of the Association of Official Analytical Chemists [21]. Moisture was determined by drying a sample in a freeze dryer overnight and then in a 105 °C convection oven for 24 h. Total nitrogen and carbon contents were measured following the Dumas method using an elemental combustion system (Costech Analytical Technologies Inc., Valencia, CA, USA), with crude protein estimated by N × 6.25. Ash was measured in a muffle furnace at 600 °C for 6 h [21].

### 2.2. Feeding Trial II

There were 7 feeding treatments, including: A-R, feeding *Artemia* at 50% of estimated saturation for 4 weeks followed by two weeks of feeding with the commercial diet B (treatment 1); A2 two weeks of *Artemia* feeding (treatment 2) or AB2, a combination of *Artemia* and commercial diet B (treatment 3) followed by two weeks of commercial diet B; A3, three weeks of *Artemia* feeding (treatment 4) or a combination of *Artemia* and commercial diet B (treatment 5) followed by 3 weeks of commercial diet B; and A4, four weeks of *Artemia* feeding (treatment 6) or a combination of *Artemia* and commercial diet B (treatment 7) followed by 2 weeks of commercial diet B. The actual feeding was presented in Table 2. The preparation of *Artemia* and fish maintenance followed the same protocols used in feeding trial I.

Lake sturgeon larvae (12 DPH) were randomly assigned to one of the 7 treatments (Table 3) with 3 replicated tanks per treatment and 120 fish (average body weight, 34 ± 1.8 mg, n = 21) per tank. *Artemia* was fed at 9:00, 11:00, 13:00, and 15:00 h, and the commercial feed (Otohime B1) was fed eight meals daily. Water temperature was maintained between 21.5–23.2 °C, dissolved oxygen > 7.6–8.1 mg/L, pH 7.5–7.9, and TAN < 0.08 mg/L. The treatments fed A2 and AB2 were terminated at the end of 4 weeks because the overall mortality was 50% or greater. During the 6-week feeding trial, all larvae in each tank were batch-weighed weekly, and feeding rates were adjusted according to fish growth (Table 2). The final body weight and survival of fish from each tank were determined following the same method described in Feeding trial I.

### 2.3. Feeding Trial III

Sturgeon larvae (27 DPH) were raised for 4 weeks with either *Artemia* or a combination of *Artemia* and the commercial diet used in the last two trials (2:1 ratio) following a similar protocol described in feeding trial II. Then, all fish were transitioned to the commercial diet for two more weeks. Water temperature was maintained at 20.2–22.3 °C, dissolved oxygen 7.8–8.1 mg/L, pH was 7.5–8.0, and TAN was <0.02 mg/L.

At the end of the 6-week trial, the fish were exposed to acute hypoxic stress. Fish were withheld from feeding and then transferred to 6 cages (width × length × height = 10 cm × 10 cm × 6 cm). Each cage held six fish, and three cages were assigned to each dietary treatment. The water temperature was similar to that in the previous culture system. The fish were acclimated to the system for 6 h, and then dissolved oxygen was decreased by injecting nitrogen gas. The level of dissolved oxygen was decreased from 8 mg/L to 2.3 mg/L at a rate of 1 mg/L hourly and mortality was monitored.

### 2.4. Data Analysis

Results were subjected to a one-way analysis of variance with significance at *p* < 0.05, with pairwise comparisons between different treatment means performed with Tukey’s studentized range (HSD) test. Tests for assumptions of normality and homogeneity of variance were performed using Shapiro–Wilk and Levene’s tests, respectively. Data violating either assumption (*p* < 0.05) were log-transformed. A second-order polynomial regression analysis was applied to estimate the relationship between the feeding duration and body weight increase (Trial I). Results for Trial III were subjected to *t*-test to compare responses of two diet treatments (A and A + B) (*p* < 0.05).

## 3. Results

### 3.1. The Potential of Substituting Artemia with Formulated Feed

The growth performance (Trial I) of lake sturgeon larvae fed different diets during the first week is shown in Table 4. The larvae fed solely the formulated diets (treatment B and L) showed significantly (*p* < 0.05) lower survival in comparison to the larvae fed *Artemia* (A) or the combinations treatment (A + B and A + L), which showed 99 to 100% survival. Feeding of the sole formulated diet generally showed poor performance in weight gain, feed conversion ratio, and protein efficiency ratio in comparison to feeding of *Artemia* or the combinations. In addition, the fish-fed diet B or L had low locomotor activity (observation) and thus these two treatments were terminated at the end of the first week.

**Figure 1:**
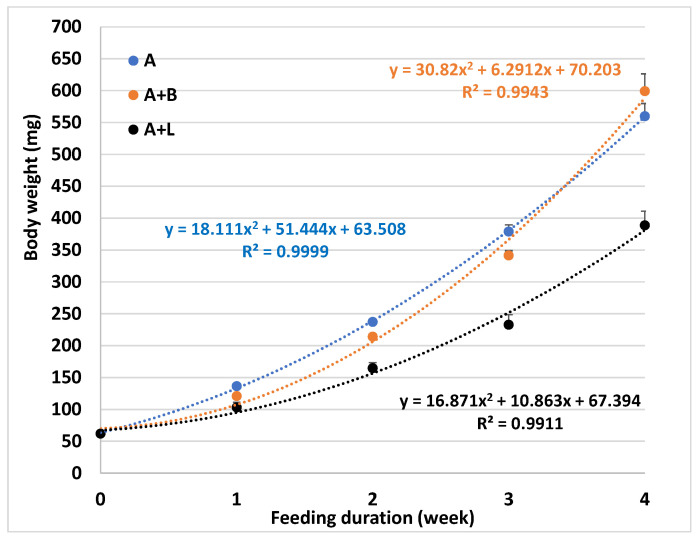
Average body weight (n = 3) of sturgeon fed different test diets during a 4-week feeding (Trial-I). A, *Artemia*; A + B, combined feeding of *Artemia* and commercial diet. A + L, combined feeding of *Artemia* and lab diet.

At the end of the 4th week feeding trial (Trial I), the survival of larvae was similar among the three remaining treatments (Table 5). The fish fed with treatment A or A + B had a similar weight gain and body weight, but those fed treatment A + L had significantly lower growth than the fish fed diet A (Table 5). The feed conversion ratio was the lowest and the protein efficiency ratio was the highest for fish fed diet A, followed by those fed A + B and then A + L. A significantly positive correlation was observed between fish weight and feeding duration for all three groups of fish (Figure 1). Although the final body weight was similar between fish-fed A and A + B, based on the regression model calculation it is likely that the fish fed the A + B will be larger than the fish fed A if feeding had continued (Figure 1). The moisture content was significantly lower, but the ratio of C/N was higher in fish fed A + B when compared to those fed A or A + L. The lowest level of ash was detected in the fish fed with A + L and the highest level was measured in the A-fed fish. No significant difference was observed in the condition factor, protein level, and RNA/DNA ratio of whole fish in response to different feedings.

### 3.2. Effect of Different Feedings on the Adaptation of Lake Sturgeon to Formulated Feed

**Figure 2:**
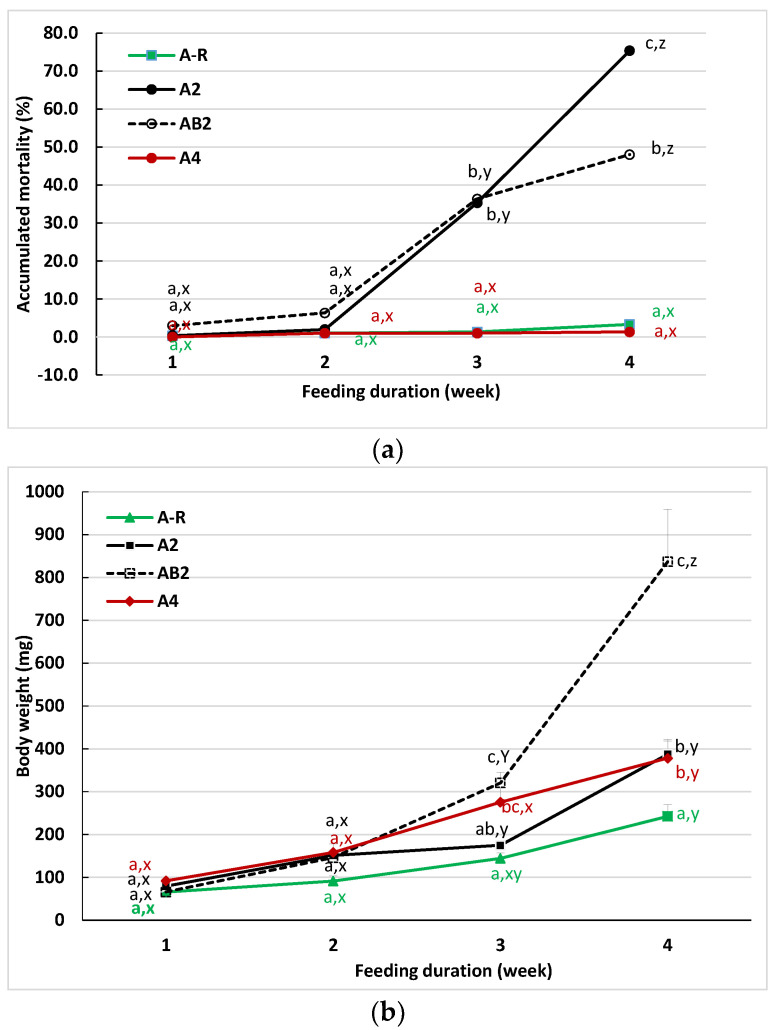
Accumulated mortality (**a**) and average body weight (**b**) of sturgeon fed with different dietary strategies for four weeks (Trial II). Data are presented as the mean of three replications. Means within the same feeding time are compared using letters a, b, and c to indicate significant differences among feeding treatments determined by Tukey’s HSD test (*p* < 0.05). Letters x, y, and z compare the difference of different sampling times within the same feeding treatment. See the footnote of Table 3 for the information on feeding treatments.

The results (Trial II) presented in Figure 2 compared the mortality and body weight of sturgeon-fed *Artemia* for 4 weeks (A-R and A_4_), sturgeon-fed *Artemia* (A_2_), or combined feeding (*Artemia* and the commercial diet, AB_2_) for 2 weeks before changing to feeding solely on the commercial diet only during weeks 3 and 4. Sturgeon raised in treatment A-R and AB_2_ were fed only 50% of *Artemia* that was provided to A_2_ or A_4_. Despite different feedings, mortality (Figure 2a) and body weight (Figure 2b) were similar among all of the treatments at the end of the 2nd week. During weeks 3 and 4, the mortality remained low (1.3 to 3.3%) for the fish continually fed with *Artemia* (treatments A and A-R). This agrees with the results observed in Trial I, which showed that *Artemia* feeding is sufficient to maintain survival during this early life stage. A reduced feeding of *Artemia* (A-R) led to significantly reduced growth fish during the third and fourth weeks when compared to the A_4_ fish. A significant increase in mortality occurred in fish previously fed with either *Artemia* (A_2_) or combined feeding (AB_2_) when their feed switched to the commercial diet at the end of the second week (Figure 2a). The mortality and body weight of fish were similar between these two treatments at the end of third week (the first week after changing feed), but at the end of the fourth week, the mortality was significantly higher and body weight was lower in treatment A_2_ than those observed in treatment AB_2_. The fish from the A-R treatment were smaller than any of the other feeding treatments.

These results indicate that co-feeding *Artemia* with the commercial feed should benefit sturgeon larvae weaning to the commercial diet. However, the timing of this transition is important for achieving the best results. Based on this study, weaning off *Artemia* at the end of 2nd week led to about 50% mortality for combined-fed fish and over 70% mortality for *Artemia*-fed fish. This suggests that complete weaning from *Artemia* two weeks after exogenous feeding begins is too early.

**Figure 3:**
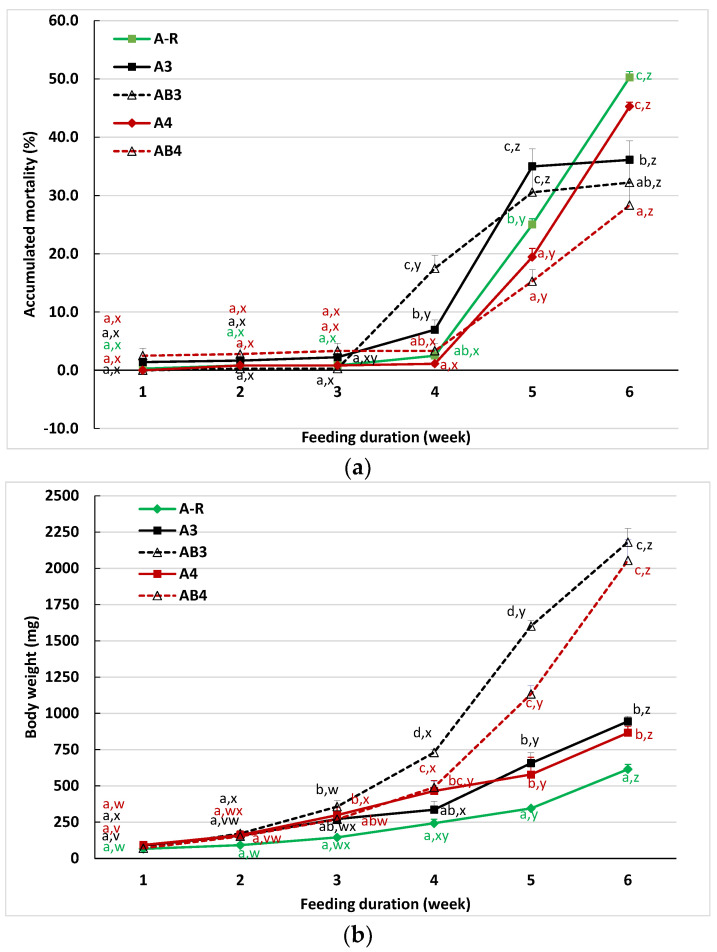
Accumulated mortality (**a**) and average body weight (**b**) of sturgeon fed with different dietary strategies for six weeks (Trial II). Data are presented as the mean of three replicates. Means within the same feeding time were compared using letters a, b, and c to indicate significant differences among feeding treatments determined by Tukey’s HSD test (*p* < 0.05). Letters x, y, and z compare the difference of different sampling times within the same feeding treatment. See the footnote of Table 3 for the information on feeding treatments.

For fish weaned at 3 or 4 weeks, mortality was low for all treatments before the fish were completely weaned to the commercial diet (Figure 3a). At the end of the sixth week, morality was the highest for A-R and A_4_. The mortality of fish from A_3_ and AB_3_ treatments was similar and reached a plateau two weeks after the fish were weaned to the commercial diet. The lowest mortality was observed with the AB4 feeding, but this was not statistically different from the mortality of AB_3_.

The differences in body weight among treatments increased with the duration of feeding (Figure 3b). No difference was observed during the first two weeks. At the end of 3rd week, the body weight was the highest for AB_3_ or AB_4_ fish but was not statistically different for the A_3_ or A_4_ fish. From weeks 4 to 6, the weight of AB_3_ and AB_4_ increased rapidly and reached about triple the weight of A_3_ and A_4_. The lowest body weight was observed for the fish from A-R feeding. In addition, body weight was not different between AB_3_ and AB_4_ or A_3_ and A_4_ treatments.

### 3.3. Effect of Different Feedings on Hypoxia Tolerance

**Figure 4:**
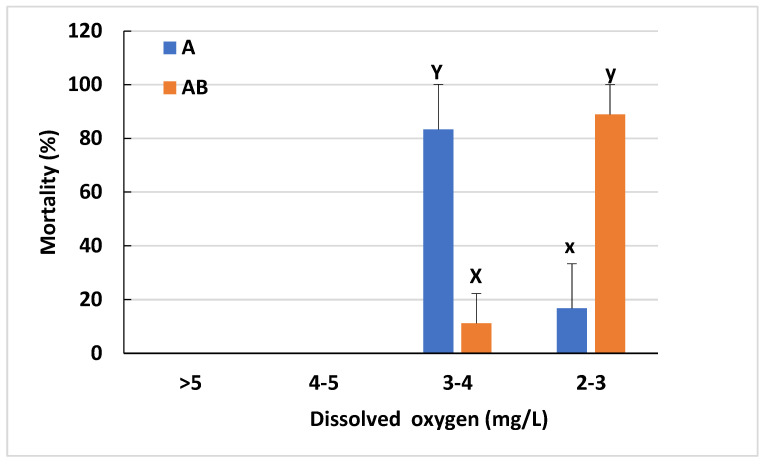
Mortality of sturgeon challenged by acute hypoxia after 6-week feeding (Trial III). Data were presented by means of mortality based on survival fish from three replications. Different letters indicated the significant difference between the two dietary treatments, with X and Y comparing the mortality that occurred at 3–4 mg/L dissolved oxygen: x and y comparing the mortality at 2–3 dissolved oxygen. A: sturgeon fed *Artemia* for 4 weeks before weaning to a commercial diet. AB: sturgeon co-fed Artemia and the commercial diet for 4 weeks before weaning to a commercial diet.

The mortality of sturgeon exposed to hypoxia (Trial III) is presented in Figure 4. Our results show that larvae fed exclusively on *Artemia* before being weaned to the commercial diet, had >80% mortality when the dissolved oxygen was between 3 and 4 mg/L. In contrast, mortality was only 11% in fish fed with 1/3 *Artemia* plus 2/3 commercial diet before weaning. The remaining fish in the two treatments all died when dissolved oxygen was reduced below 3 mg/L. This indicates that larvae co-fed with *Artemia* had better tolerance to hypoxia.

## 4. Discussion

The results of this study demonstrated that neither the commercial diet nor the lab diet alone could support good survival of larvae at early life stages up to 49 DPH. A combination of the commercial diet (B) and a reduced level of *Artemia* (50% ratio of the *Artemia* group) did not cause a negative impact on the survival and the growth of the sturgeon. This finding suggests that the commercial diet can partially replace *Artemia* to support the nutrients required by the larval fish. In a previous study by Lee et al. [12], lake sturgeon juveniles obtained good growth when fed this lab-made diet, verifying that it meets the nutritional and physical quality requirements for juvenile fish. Low survival and poor growth of the larvae reared solely on the formulated diets assessed in the current study are comparable to previous reports [9,10,22,23]. DiLauro et al. [9] commented that poor acceptance of an artificial diet by the larvae may have been attributed to a lack of imprinting. This may partially explain why the inferior performance of sturgeon larvae fed the lab-made diet compared to the fish fed the commercial diet, which had been used to condition the larvae for one week before the feeding trial. On the other hand, based on the proximate compositions of the three diets, the lab-made diet had lower protein, lipid, and ash contents than the other two diets (Table 2). A much higher n-6 fatty acid was measured in the lab diet than in the other two diets. Thus, the difference in the nutritional composition of the lab-made diet and the commercial diet may explain the poor performance of larvae fed the lab diet. The commercial diet and *Artemia* were similar in proximate composition but possess different fatty acid profiles. The level of linolenic acid was higher in *Artemia*, but eicosapentaenoic acid (EPA, 20:5n-3) and docosahexaenoic acid (DHA, 22:6n-3) were lower when compared to the commercial diet. The lab-made diet also had low levels of EPA and DHA compared to the commercial diet. Although the nutritional requirement of lake sturgeon has not been investigated, both n-3 and n-6 long-chain unsaturated fatty acids as well as highly unsaturated fatty acids are required by other sturgeon species [24]. Further investigation into the dietary fatty acid requirements of lake sturgeon larvae is warranted.

A similar protein level in larval fish was observed in the fish larvae across the different treatments, indicating that protein supply should be similar from the three feeding treatments. The RNA/DNA ratio, an indicator of protein biosynthesis, has been used as an indicator to determine growth rates in larval fish [20,25]. It is also a biomarker of nutritional status of fish in response to feeding [13,26,27,28]. The similar ratio of RNA/DNA of sturgeon fed the diet A, A + B, or A + L agrees with the results for growth and protein content, which were similar among all treatments. This indicates that the three feeding regimes provided sufficient nutrition to support their growth at this stage. Lipid is one major energy reserve present in a fish body and provides an important indication of the fish’s nutritional status. The analysis of lipid content involves a time-consuming process and requires an adequate sample size to achieve reliable results, which is challenging when a limited sample is available. In general, lipid molecules contain a very low number or no nitrogen with carbon as their major element. Thus, C/N ratios in bulk tissue positively correlate with lipid levels. This provides the rationale for applying C/N ratios to predict the lipid content of fish in previous studies [29,30,31]. In our previous study, we also observed a positive relationship between lipid content and C/N ratios in lake sturgeon [13]. Thus, in the current study, based on the results of C/N ratios of whole fish tissue we predict that the lipid content was lower in fish fed *Artemia* or *Artemia* + Lab diet than in *Artemia* + Commercial diet, suggesting that the feeding A + B resulted in higher energy reserves. The difference in fish ash content reflected the dietary ash levels, with the highest in diet A and the lowest in L. Besides the nutritional quality, physical qualities such as water stability are critical because small particle sizes increase the risk of nutrient leaching [32]. Based on our observations, the lab-made diet had poorer water stability than the commercial diet. Consequently, the nutrient availability of the lab-made diet may have been reduced as a result. This may explain the decreased protein efficiency ratio, higher feed conversion ratio, and poorer growth of sturgeon fed the A + L combined diet. The challenges are nutrient leaching especially difficult to overcome in slow-feeding fish such as sturgeon.

We observed that feeding solely *Artemia* to the sturgeon can support survival and growth during the first 4 weeks of exogenous feeding. Similar findings were reported by Bauman et al. [10] and Valentine et al. [11] through 14 days and 35 days post-exogenous feeding, respectively. The results of these two studies and our current findings support the rationale that the nutritional profile of *Artemia* nauplii can be used as a baseline for developing larval feed for lake sturgeon. However, it is particularly critical to note that the nutritional requirements required to support growth and physiological function may not parallel the requirements for optimizing stress tolerance [33]. As we discussed above, the commercial diet contained a high level of EPA and DHA, which was low or not present in *Artemia*. Thus, compared to *Artemia*-feeding larvae, sturgeon larvae under combined feeding might accumulate a higher level of EPA or DHA, which have been found to improve digestive activity, survival, and growth of lake sturgeon, and the tolerance of larval fish to hypoxia in different fish species, such as Adriatic sturgeon (*Acipenser naccarii*), Dove Sole (*Solea solea*) and European eel (*Anguilla anguilla*), owning to lowered metabolic rate and oxygen consumption in fish fed these fatty acids [34,35,36,37]. This may partially explain why combined feeding enhanced the tolerance of lake sturgeon to the hypoxia challenge in the current study. In contrast, the *Artemia*-fed sturgeon might not accumulate substantial EPA and DHA to obtain this benefit because of only being on the commercial diet for two weeks. Further investigation is needed to test this hypothesis and understand mechanisms for lake sturgeon tolerance to hypoxia.

In the current study, we did not investigate the digestive enzyme activities of the gastrointestinal tract, which play pivotal roles in fish feeding. The composition and overall activity of digestive enzymes have been observed to change with oncogenic stages and are also influenced by exogenous feeding [34,38,39,40,41]. For example, during Stelate sturgeon (*Acipenser stellatus*) larval development, the activity of trypsin, chymotrypsin, and amylase was decreased while the activity of pepsin, alkaline phosphatase, and lipase increased, suggesting that these larval fish become dependent on lipid to meet their metabolic energy requirements but spare protein for maintenance and tissue synthesis, thus appropriate lipid supply from feeding at this stage is necessary to optimize their growth [40]. Furthermore, the appearance and activity of digestive enzymes are important factors supporting the diet weaning of larval fish. It was reported that about 2 weeks post exogenous feeding (the first-month post-hatching) in Adriatic sturgeon *(Acipenser naccarii),* digestive amylase and protease activities reached stabilization with a fully developed digestive structure [39]. For Persian sturgeon (*Acipenser persicus*), the maturation of their digestion functions was found to be achieved by 19–24 days post-hatching when diet weaning is recommended [38]. Recent findings by Yoon et al. [34] also discovered that enriched feeding with DHA resulted in increased lipase activity and survival of lake sturgeon during the weaning period. Thus, it is highly likely that dietary EPA and DHA have made a significant contribution to enhancing the digestive enzyme activity and thus growth and survival of the lake sturgeon co-fed with the commercial diet in the current study [41].

## 5. Conclusions

The current study demonstrates that feeding solely on *Artemia* can support good survival and growth of lake sturgeon during 28 days of post-exogenous feeding, but co-feeding with the commercial diet benefits weaning to a formulated diet based on growth, survival, and tolerance to hypoxia. Co-feeding of lake sturgeon larvae for 3 or 4 weeks after initial exogenous feeding is recommended before lake sturgeon larvae are weaned to the commercial diet to receive these benefits. Partial substitution of *Artemia* by the commercial feed did not cause adverse impacts on the growth and survival of the lake sturgeon. Our results indicate that nutrient enrichment should be considered in live feed commonly used for lake sturgeon rearing, including *Artemia* (lacking eicosapentaenoic acid and docosahexaenoic acid) to enhance fish health and tolerance to stress. A successful transition to alternative feed is dependent on both feed quality and the capacity of digestive enzymes, which warrants future research. The use of formulated feeds should offer a cost-effective approach to lake sturgeon larviculture, but more research is needed to address the influences on fish health, behavior, and stress tolerance, considering that lake sturgeon is cultured to support population conservation efforts.

## Figures and Tables

**Table 1:** Experimental treatments of Feeding trial I.

Treatment	Feeding Rate (% BW) during the 4 Weeks			
	1	2	3	4
*Artemia* (A)	10	7.5	6.5	4.8
Commercial diet (B)	20	No more feeding		
Lab diet (L)	20	No more feeding		
A + B	A: 5, B: 10	A: 3.75, B: 7.5	A: 3.25, B: 6.5	A: 2.4, B: 4.8
A + L	A: 5, L: 10	A: 3.75, L: 7.5	A: 3.25, L: 6.5	A: 2.4, L: 4.8

The initial average weight of lake sturgeon larvae was 62 mg ± 1.1 (n = 15, 21-day post-hatch).

**Table 2:** Nutritional composition of test diets for Feeding trial I.

		Test Diet	
Analysis	*Artemia nauplii*	Commercial Feed (Otohime B)	Lab-Made Diet
Proximate composition (g/kg, dry matter basis)			
Moisture	946	65	110
Protein	513	551	491
Lipid	164	180	139
Ash	98	129	76
Major fatty acid (g/kg of total fat)			
16:0	113.4	176.6	187
16:1	31.9	41.8	49.7
18:0	40.2	22.8	56.2
18:1 n-9	187.0	100.4	138.3
18:2 n-6	56.3	53.1	218.1
18:3 n-3	308.9	44.2	47
20:5 n-3	14.9	123.3	54.5
22:6 n-3	0.3	107.8	38.1

**Table 3:** Feeding rate (based on a percentage of body weight) for lake sturgeon during 6 weeks of feeding (Feeding trial II).

Dietary Treatment			Feeding Duration (Week)			
	1	2	3	4	5	6
	Feeding Rate ^8^					
A-R ^1^	10 A	5 A	3 A	3 A	9 B	9 B
A_2_^2^	20 A	10 A	9 B	9 B		
A_3_^3^	20 A	10 A	6 A	9 B	6 B	6 B
A_4_^4^	20 A	10 A	6 A	6 A	6 B	6 B
AB_2_^5^	10 A + 10 B	5 A + 10 B	9 B	9 B		
AB_3_^6^	10 A + 10 B	5 A + 10 B	3 A + 6 B	9 B	6 B	6 B
AB_4_^7^	10 A + 10 B	5 A + 10 B	3 A + 6 B	3 A + 6 B	6 B	6 B

^1^ A-R: fish were fed to 50% of the estimated satiation with artemia for our weeks before they were transited to feeding the commercial diet (B). ^2^ A_2_: fish were fed to the estimated satiation for **two** weeks with *artemia* (A) before they were switched to the commercial diet (B). This treatment was finished at the end of the 4th week. ^3^ A_3_: fish were fed to the estimated satiation for **three** weeks with *artemia* (A) before they were switched to the commercial diet (B). ^4^ A_4_: fish were fed to the estimated satiation for **four** weeks with *artemia* (A) before they were switched to the commercial diet (B). ^5^ AB_2_, ^6^ AB_3_, and ^7^ AB_4_: fish were co-fed with *artemia* and the commercial diet (B) for 2, 3, or 4 weeks, respectively, before they were switched to the commercial diet (B). The treatment AB_2_ was finished at the end of the 4th week. ^8^ The numbers before each capital letter represent the feeding rate (based on % body weight) and the capital letters represent two different diets (A for *artemia* and B for the commercial diet). The initial body weight of the fish was 34 ± 1.8 mg, n = 21, 14-day post-hatch.

**Table 4:** Growth performances of lake sturgeon larvae fed the different diets for one week (Trial I) ^1^.

Treatment ^2^					
Measurement	A	B	L	A + B	A + L
Survival (%)	100.0 ± 0.0 ^A^	78.2 ± 2.7 ^B^	80.9 ± 7.2 ^B^	99.1 ± 0.9 ^A^	99.1 ± 0.9 ^A^
Final body weight (mg)	137 ± 3 ^A^	93 ± 6 ^C^	81 ± 1 ^C^	121 ± 3 ^AB^	103 ± 8 ^BC^
Weight gain ^3^ (%)	120.2 ± 4.3 ^A^	49.3 ± 10.2 ^C^	29.9 ± 1.9 ^C^	95.1 ± 5.6 ^AB^	66.4 ± 12.9 ^BC^
Feed conversion ratio ^4^	0.4 ± 0.0 ^C^	2.6 ± 0.7 ^AB^	3.6 ± 0.2 ^A^	0.8 ± 0.1 ^C^	1.2 ± 0.3 ^BC^
Protein efficiency ratio ^5^	4.74 ± 0.17 ^A^	0.74 ± 0.15 ^C^	0.53 ± 0.03 ^C^	2.07 ± 0.12 ^B^	1.65 ± 0.32 ^B^

^1^ Means ± SEM (n = 3) with different superscripts within each row are significantly (*p* < 0.05) different by Tukey’s studentized range test. ^2^ Treatment: A, *Artemia*; B, commercial diet; L, lab diet; A + B, a combination of A and B; A + L, a combination of A and L; Treatment B and L were terminated at the end of the first week feeding due to the high mortality observed in these treatments. ^3^ Weight gain (%) = 100 × (final body weight (mg) − initial body weight (mg))/initial body weight (mg). ^4^ Feed conversion ratio = Dry feed weight provided (mg)/weight gain (mg). ^5^ Protein efficiency ratio = Fish weight gain (mg)/protein provided (mg).

**Table 5:** Growth performances and whole-body proximate composition of lake sturgeon fed with different diets for 4 weeks (Trial I) ^1^.

Treatment ^2^			
	A	A + B	A + L
**Growth performance**			
Survival (%)	96.7 ± 0.7	94.7 ± 2.9	92.7 ± 1.8
Final body weight (mg)	560 ± 20 ^A^	599 ± 27 ^A^	389 ± 22 ^B^
Weight gain (%)	803.4 ± 31.8 ^A^	866.8 ± 43.6 ^A^	527.3 ± 35.4 ^B^
Condition factor	0.34 ± 0.01	0.34 ± 0.00	0.33 ± 0.00
Feed conversion ratio	0.5 ± 0.0 ^C^	0.7 ± 0.0 ^B^	0.8 ± 0.0 ^A^
Protein efficiency ratio	3.83 ± 0.11 ^A^	2.55 ± 0.10 ^B^	2.22 ± 0.08 ^B^
**Proximate composition**			
Moisture (g/kg)	895 ± 3 ^A^	880 ± 3 ^B^	900 ± 3 ^A^
Crude protein (g/kg)	78 ± 1	83 ± 1	76 ± 3
Crude ash (g/kg)	14 ± 1 ^A^	13 ± 0 ^AB^	11 ± 1 ^B^
C/N ratio	3.74 ± 0.05 ^B^	4.25 ± 0.13 ^A^	3.70 ± 0.02 ^B^
RNA/DNA ratio	1.12 ± 0.09	1.19 ± 0.08	1.26 ± 0.14

^1^ Means ± SEM (n = 3) with different superscripts within each row are significantly (*p* < 0.05) different by Tukey’s HSD test. ^2^ Definition of treatment sees Table 4 for a footnote.

## Data Availability

The data presented in this study are available on request from the corresponding author.
